# Multimodal Treatment of Hepatocellular Carcinoma in Patients With Hepatitis C Virus Infection Treated With Direct-Acting Antivirals

**DOI:** 10.7759/cureus.25487

**Published:** 2022-05-30

**Authors:** Adriana Mercan-Stanciu, Teodora Isac, Razvan Rababoc, Daniel Rusie, Letitia Toma, Ileana Adela Vacaroiu, Raluca Tulin, Elena Laura Iliescu

**Affiliations:** 1 Department of Internal Medicine II, Fundeni Clinical Institute, Bucharest, ROU; 2 Department of Internal Medicine II, Carol Davila University of Medicine and Pharmacy, Bucharest, ROU; 3 Department of Internal Medicine, Carol Davila University of Medicine and Pharmacy, Bucharest, ROU; 4 General Surgery, Bucharest Emergency Clinical Hospital, Bucharest, ROU; 5 Department of Nephrology, Faculty of Medicine, Carol Davila University of Medicine and Pharmacy, Bucharest, ROU; 6 Department of Anatomy and Embryology, Carol Davila University of Medicine and Pharmacy, Bucharest, ROU; 7 Department of Endocrinology, Agrippa Ionescu Emergency Hospital, Bucharest, ROU

**Keywords:** direct-acting antivirals, liver transplantation, sorafenib, hepatitis c virus infection, hepatocellular carcinoma

## Abstract

Background: Direct-acting antivirals (DAAs) opened a new era in the management of hepatitis C virus (HCV)-associated liver disease. However, hepatic cancer screening should not be stopped after obtaining a sustained virologic response (SVR). Current guidelines offer several treatment options for hepatocellular carcinoma (HCC), mainly depending on its stage and the extent of liver disease, including tumor resection, liver transplantation (LT), radiofrequency ablation (RFA), transarterial chemoembolization (TACE), and systemic agents. This article provides an overview of treatment modalities for hepatocellular carcinoma and associated survival rates based on the experience of the Internal Medicine Center at Fundeni Clinical Institute while bringing into light previous medical research.

Methods: We included 59 patients with a personal history of hepatitis C virus infection, diagnosed with hepatocellular carcinoma at least one year after achieving a sustained virologic response through direct-acting antivirals. The albumin-bilirubin (ALBI) score and Barcelona Clinic Liver Cancer (BCLC) classification were assessed in each case, and all patients were treated accordingly. The subjects were monitored by liver function tests, tumor markers, blood cell count, coagulation profile, and imaging explorations. We investigated the Eastern Cooperative Oncology Group (ECOG) performance status, the response to applied treatments, and survival.

Results: Cirrhotic patients and multinodular tumor patterns were predominant. Most patients only experienced one therapeutic procedure, while the rest of the study group went through multiple treatment modalities (2-4), with a better outcome in terms of survival parameters. A large proportion presented with disease progression despite the therapeutic measures applied. A total of two liver transplants were performed, resulting in a 12-month disease-free period among these patients. The presence of diabetes mellitus (DM), multinodular disease, alpha-fetoprotein (AFP) over 300 ng/mL, and tumor dimension over 6 cm indicate poor overall survival. Both overall survival and progression-free survival were better in subjects who presented complete responses (CR) to HCC treatment. In patients undergoing a single intervention, the best overall survival was associated with surgical resection and RFA.

Conclusion: The multimodal treatment of hepatocellular carcinoma represents the best approach, in order to maintain patients on the waiting list for liver transplantation. In hepatitis C virus infection, viral clearance is important to obtain. At the same time, particular attention should be paid to liver cancer screening even after obtaining a sustained virologic response.

## Introduction

Hepatocellular carcinoma (HCC) is known to be the fourth major cause of cancer-related mortality worldwide, and the World Health Organization recently revealed that more than one million individuals are expected to die due to hepatic cancer in 2030 [1-3]. For many years, viral hepatitis has been one of the main risk factors for HCC occurrence. In particular, hepatitis C virus (HCV) is associated with an up to 20-fold increase in HCC development [4]. Furthermore, efforts should be made in order to identify new biomarkers for early diagnosis and therapy in specialized medical centers [5,6].

Although the use of direct-acting antivirals (DAAs) opened a new era in the management of HCV-induced liver disease, the relationship between DAAs and HCC was associated with numerous controversies. Several authors suggest that interferon-free regimens lead to faster tumor growth, while other researchers found no link between HCC occurrence and DAA therapy [7-9]. The essential factor for attempting a curative procedure is represented by an early diagnosis of hepatocellular carcinoma; thus, HCC screening is mandatory in susceptible patients, including those who have already achieved a sustained virologic response (SVR). The Barcelona Clinic Liver Cancer (BCLC) staging system uses both clinical and imagistic criteria, linking HCC stage, patient’s performance status, and comorbidities, in order to determine the appropriate therapeutic strategy for each patient [10]. The BCLC system can be used to recognize early-stage HCC, in which cases curative therapies may successfully be applied.

The aim of the study was to analyze survival parameters and treatment response in patients with HCC who previously received DAA treatment while comparing the results with those already reported in literature among subjects with no history of DAA treatment.

## Materials and methods

We conducted a retrospective observational study on 59 patients who had a prior history of HCV infection, for which they received treatment with direct-acting antivirals (either ritonavir-boosted paritaprevir/ombitasvir and dasabuvir or ledipasvir/sofosbuvir), in accordance to the National Healthcare Program available at the moment. Each patient obtained a sustained virologic response, defined as undetectable HCV ribonucleic acid (HCV-RNA) at 12 weeks after ending DAA treatment. The subjects were admitted to the Internal Medicine Center at Fundeni Clinical Institute between January 2015 and December 2019. The study was performed with approval from the Hospital Ethical Committee. Informed written consent was taken from all the participants, and all their records were confidential.

The inclusion criteria were documented de novo hepatocellular carcinoma and personal history of HCV chronic infection, with SVR after DAAs.

We excluded from the study patients with other concomitant or previous cancers, autoimmune liver disorders, hepatitis B virus (HBV) or human immunodeficiency virus (HIV) coinfection, and HCV who were not treated with DAAs.

We applied the BCLC classification to each patient. The very early stage (BCLC 0) and early-stage (BCLC A) HCC included asymptomatic patients with preserved liver function, presenting with either solitary masses or with 2-3 nodules (under 3 cm in diameter), without macrovascular invasion or extrahepatic spread. Intermediate-stage HCC (BCLC B) included patients with preserved liver function and unresectable tumors in the absence of vascular invasion or extrahepatic spread. Patients with advanced-stage disease (BCLC C) had portal invasion or extrahepatic spread, as well as mild cancer-related symptoms, while still maintaining a good liver function. Patients with terminal-stage HCC (BCLC D) had an end-stage liver function or significant cancer-related symptoms. The therapeutic options taken into consideration were systemic chemotherapy with sorafenib, transarterial chemoembolization (TACE), surgical resection, percutaneous radiofrequency ablation (RFA), and liver transplantation (LT). Subjects with BCLC D received the best supportive care. Demographic profiles were recorded. Patients’ evaluation included liver function tests, renal function monitoring, blood cell count, coagulation profile, and tumor markers such as serum alpha-fetoprotein (AFP) levels. Important comorbidities such as diabetes mellitus (DM), metabolic syndrome, and hypothyroidism were also noted and monitored, when present. The albumin-bilirubin (ALBI) score was also determined to assess liver function in patients with HCC. The cut points of the ALBI grade were as follows: ≤−2.60, ALBI grade 1; from −2.60 to −1.39, ALBI grade 2; and >−1.39, ALBI grade 3 [11].

All patients underwent conventional abdominal ultrasonography examination, as well as either contrast-enhanced ultrasonography (CEUS), computed tomography (CT), or magnetic resonance imaging (MRI), describing the number of nodules, maximum diameter, and location. We also evaluated the tumor burden score (TBS), a parameter that includes both number and size into a single variable. The cut points for the TBS were as follows: high, over 13.74; medium, 3.36-13.74; and low, less than 3.36.

We investigated the Eastern Cooperative Oncology Group (ECOG) performance status, the modified Response Evaluation Criteria in Solid Tumours (mRECIST), and the following survival parameters: disease-free survival, progression-free survival, and overall survival. Disease-free survival was measured after a certain HCC therapeutic procedure was performed resulting in complete response (CR) and was defined as the amount of time without tumor recurrence. Progression-free survival was measured from the initiation of the first HCC therapeutic procedure to HCC progression (as defined by the mRECIST). Overall survival was defined as the time between the initiation of HCC-targeted procedures and the death of any cause.

The six ECOG performance status grades are defined in Table 1 [12].

**Table 1:** Description of the ECOG performance status

Grade	Description
0	Fully active, able to carry on all pre-disease performance without restriction
1	Restricted in physically strenuous activity but ambulatory and able to carry out work of a light or sedentary nature
2	Ambulatory and capable of all self-care but unable to carry out any work activities
3	Capable of only limited self-care, confined to bed or chair more than 50% of waking hours
4	Completely disabled, cannot carry on any self-care, totally confined to bed or chair
5	Dead

The mRECIST evaluation included four types of response, as shown in Table 2 [13].

**Table 2:** Definition of mRECIST evaluation response types

Response type	Definition
Complete response (CR)	Vanishing of arterial enhancement in all lesions
Partial response (PR)	A reduction of over 30% in the sum of the diameters of all viable tumors
Stable disease (SD)	Insignificant changes concerning tumor size
Progressive disease (PD)	An expansion of over 20% in the sum of the diameters of all viable tumors

Statistical analysis was conducted using the SPSS software version 17.0 (SPSS Inc., Chicago, IL, USA). We calculated survival using the Kaplan-Meier method, while variables associated with survival in univariate analysis were included in the multivariate analysis. A p-value of less than 0.05 was considered statistically significant.

## Results

**Figure 1:**
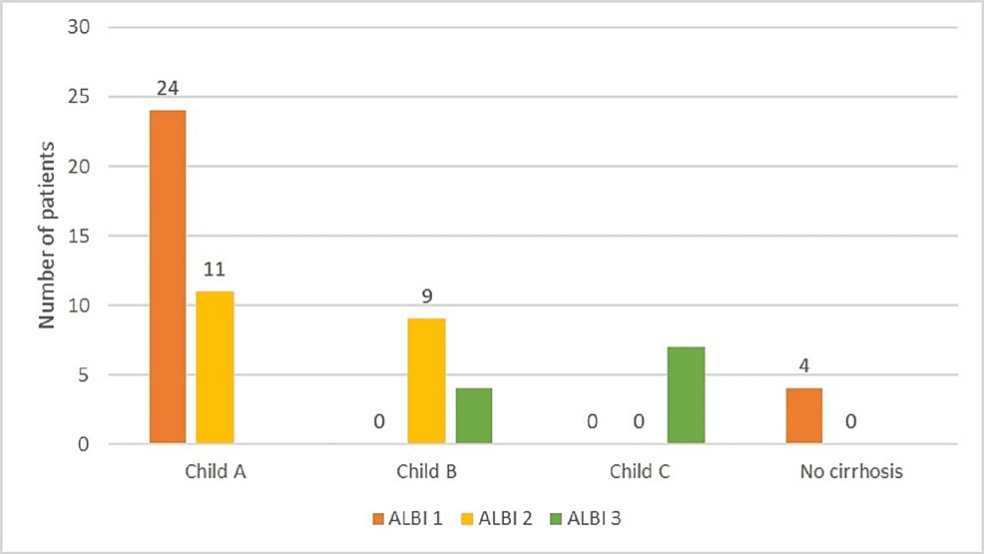
Distribution of study group by Child-Pugh class and ALBI score

We collected and analyzed data from 59 patients, most of whom were male (n = 38, almost 65%). The mean age at HCC diagnosis was 52.2 ± 20.6 years. The median time to follow-up from HCC diagnosis to death or final medical record was 17.1 months. Cirrhotic patients were predominant (93.22% of all patients), with 59.32% of the total number classified as class A Child-Pugh cirrhosis. Approximately half of the patients were identified as ALBI grade 1 (Figure 1).

**Figure 2:**
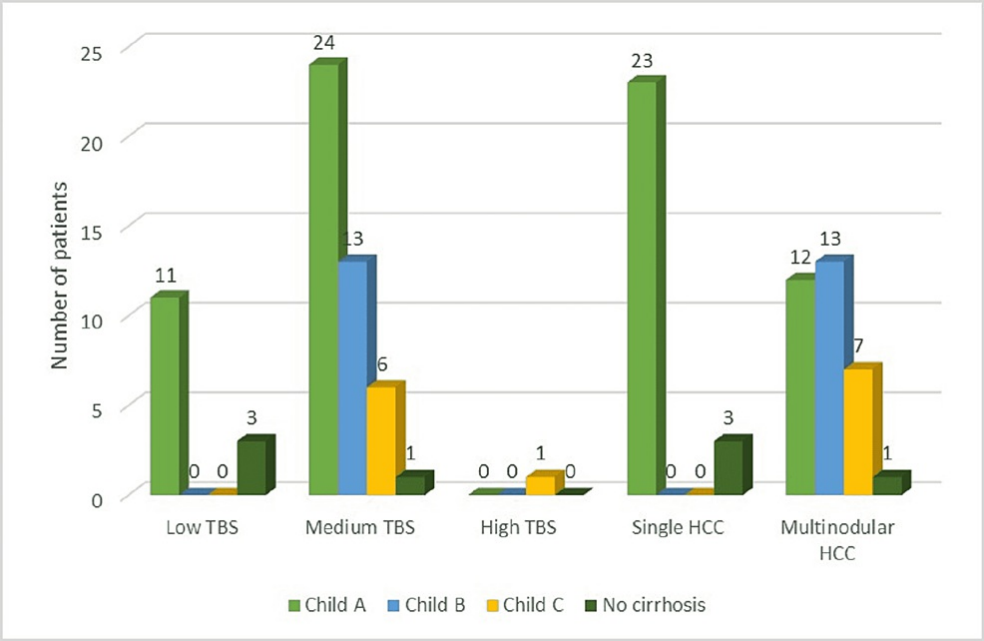
Distribution of study group by Child-Pugh class, TBS, and HCC pattern

Regarding the HCC pattern, solitary masses were found in 44% of patients, while the rest were multinodular hepatocellular carcinomas (Figure 2).

The BCLC classification resulted in the following distribution of patients: 3.39% were in stage 0, 23.72% of the patients were in stage A, 33.89% had stage B, 27.11% were in stage C, and 11.86% in stage D. All patients with BCLC stage D corresponded to ALBI grade 3.

Over 71% (42 patients) only experienced one therapeutic procedure (including four advanced-stage subjects who could only benefit from the best supportive care), while the rest of the study group went through multiple therapeutic means: 13.55% underwent two procedures, 11.86% experienced three procedures, and 3.38% (two patients) had four interventions.

**Figure 3:**
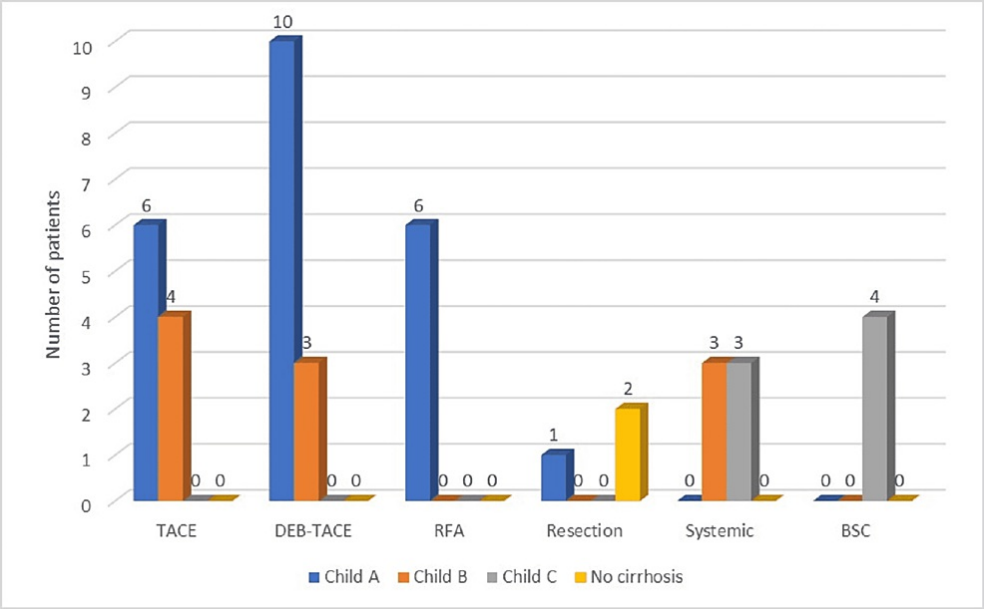
Treatment options and liver function in patients who underwent a single procedure

Out of the 42 patients who only benefitted from one intervention, surgical resection was performed in three patients (7.14%), while more than half (23 patients, 54.76%) underwent TACE: 10 patients with conventional TACE and 13 subjects with doxorubicin eluting beads transarterial chemoembolization (DEB-TACE). RFA was the chosen therapeutic measure for six out of the 42 patients (14.28%), while six other patients received systemic chemotherapy. In four cases (9.52%), the best supportive care was the only suitable choice. Patients who experienced TACE had significantly larger masses than the subjects submitted to liver resection (5.9 cm versus 7.3 cm, p < 0.001). Also, hepatic dysfunction, as shown by liver function tests and Child-Pugh classification, was more advanced in patients undergoing TACE than in those with RFA (p < 0.05) (Figure 3).

Only one of these 42 patients underwent liver transplantation after the initial chemoembolization procedure. Only two of the 59 patients included in the study underwent liver transplantation, while the other 35 patients (60%) were included on the waiting list.

A total of 17 patients benefitted from two or more treatment options. Eight patients (13.55%) had two therapeutic procedures performed: two patients with both surgical resection and systemic therapy, three patients with both TACE and DEB-TACE, and the other three patients with TACE and systemic therapy. Seven patients (11.86%) had three therapeutic procedures performed: one was subjected to RFA, followed by sorafenib and best supportive care; another patient underwent surgical resection and conventional TACE and also benefitted from systemic therapy; in two of the cases, the chosen therapeutic measures were represented by DEB-TACE, conventional TACE, and systemic therapy, while three patients underwent surgical resection and DEB-TACE and also received systemic therapy. Two patients (3.38%) had four procedures performed: one patient with RFA, TACE, DEB-TACE, and best supportive care, and the other patient with TACE, DEB-TACE, sorafenib, and best supportive care.

**Figure 4:**
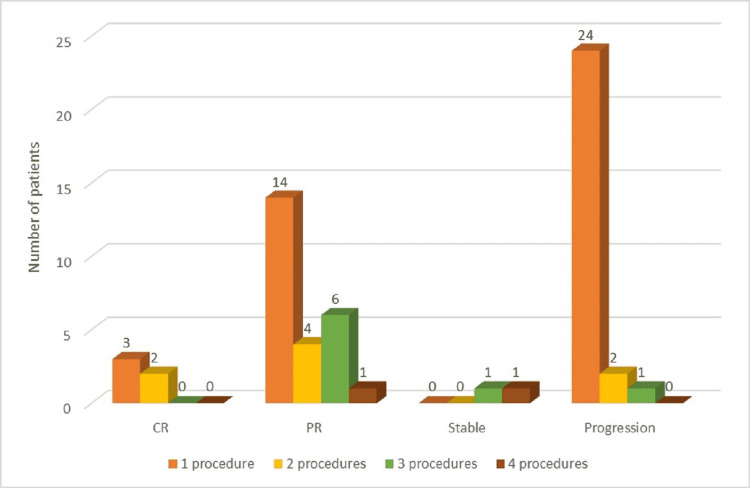
HCC treatment and mRECIST

Analyzing mRECIST resulted in five patients (8.47%) with a complete response at 12-month follow-up and 25 patients (42.37%) with only partial response, while a larger proportion (27 patients, 45.76%) presented with disease progression despite the therapeutic measures applied, and two subjects (3.38%) had insignificant changes concerning tumor size (defined as stable disease). HCC progression was more pronounced in patients who only underwent one therapeutic measure (p < 0.05). Figure 4 depicts treatment response in correlation with the number of therapeutic measures applied.

Patients with disease progression also had advanced cirrhosis and larger tumor masses at diagnosis, as well as important other comorbidities, such as diabetes mellitus (17 patients), hypothyroidism (12 patients), and metabolic syndrome (11 patients), with the proportion of these associated pathologies being higher than among patients with CR or PR.

**Figure 5:**
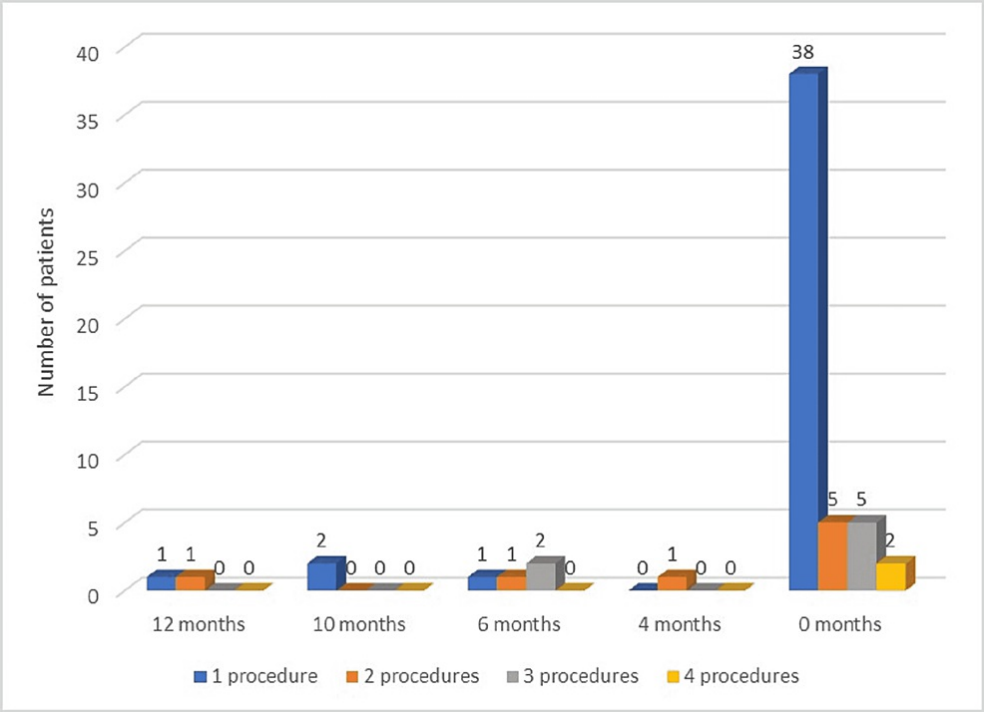
Disease-free survival according to the number of therapeutic measures applied

In terms of disease-free survival rates, our study revealed that only one patient (1.69%) had a disease-free survival period of four months, four patients (6.77%) had a disease-free survival period of six months, two patients (3.38%) presented a disease-free survival period of 10 months, and another two patients had a disease-free survival period of 12 months. It is important to mention that the patients with a 12-month disease-free period were the ones who also underwent liver transplantation. Moreover, the subjects with a 10-month disease-free period had no cirrhosis, corresponded to BCLC stage 0, and only benefitted from surgical resection. On the other hand, most patients (50 subjects, 84.74%) did not present a disease-free interval. More details regarding disease-free survival, according to the number of therapeutic measures applied, are shown in Figure 5.

**Figure 6:**
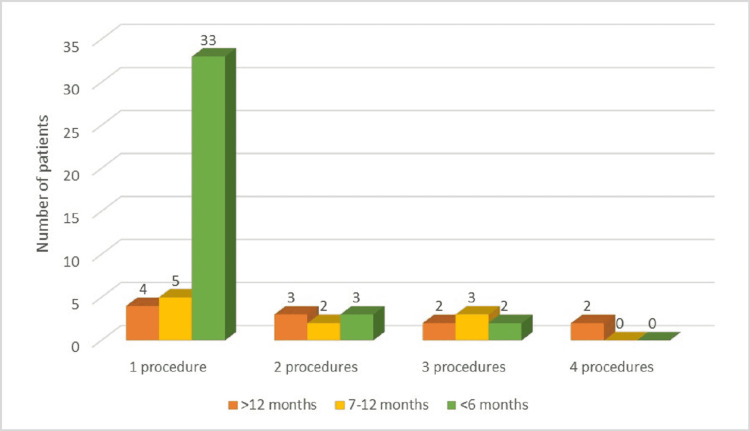
Progression-free survival (months) according to the number of therapeutic measures applied

Analysis of progression-free survival rates (Figure 6) resulted in the following distribution: under six months for 38 patients (64.40%), 7-12 months for 11 patients (18.64%), and more than 12 months for 10 patients (16.94%).

**Figure 7:**
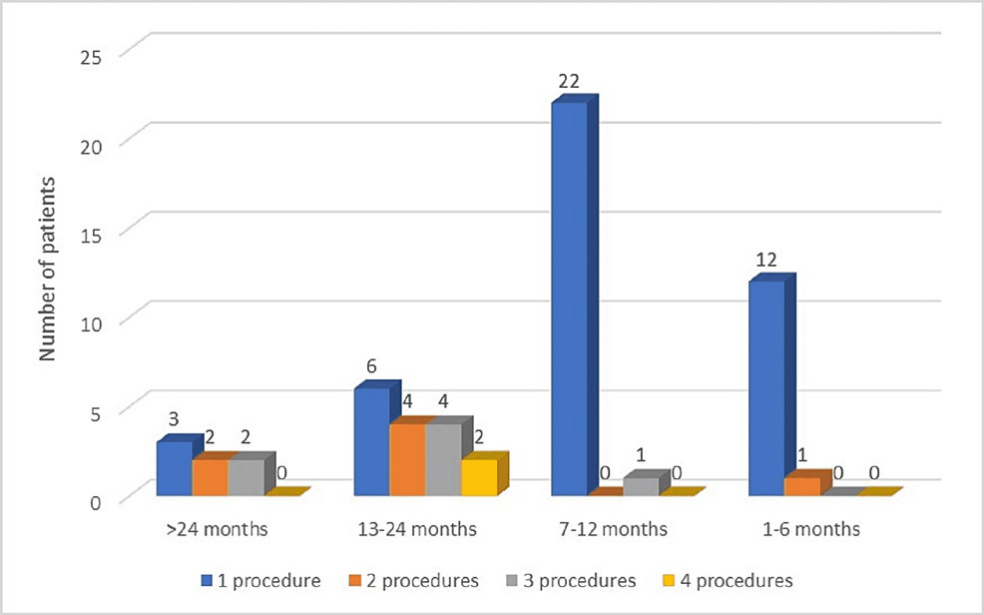
Overall survival according to the number of therapeutic measures applied

Noticeably, the use of multiple procedures resulted in higher progression-free rates. Assessment of the overall survival rates revealed the following: an overall survival rate of 1-6 months for 13 patients (22.03%), 7-12 months for 23 patients (38.98%), 13-24 months for 16 patients (27.11%), and over 24 months for seven patients (11.86%). We observed that the parameters associated with survival were improved in patients who benefitted from multiple procedures compared to individuals in whom only one therapeutic measure was performed (Figure 7).

Moreover, the presence of comorbidities (especially DM), portal hypertension, multinodular disease (with more than three tumoral masses), AFP levels higher than 300 ng/mL, high TBS, and nodule diameter larger than 6 cm indicate poor overall survival (p = 0.001).

**Figure 8:**
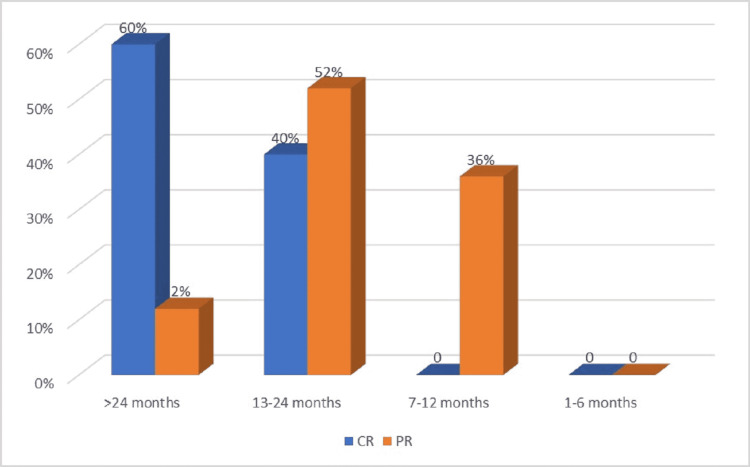
Overall survival in patients with complete response (CR) versus partial response (PR)

It is noticeable that among patients with CR, the overall survival was better (17.9 months) than in patients who only had PR (17.5 months, p = 0.04) and also better than in subjects with disease progression (14.5 months, p = 0.01) (Figure 8).

**Figure 9:**
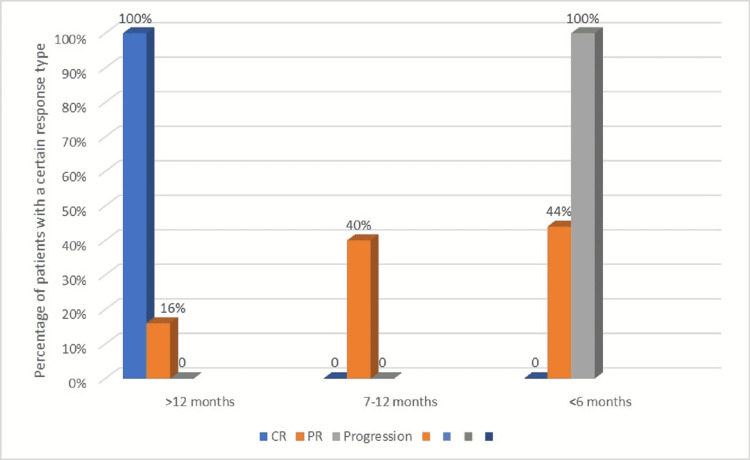
Progression-free survival in patients with complete response (CR) versus partial response (PR)

A similar observation can be made about progression-free survival: 8.8 months for patients with CR, 6.8 months for patients with PR (p = 0.03), and 2.8 months for patients with progressive disease (p = 0.01) (Figure 9).

Patients with CR had also better disease-free survival rates than both patients with PR or progressive disease (p < 0.001 in both cases).

We also assessed survival parameters in patients with a single intervention and observed that the surgical resection of HCC had a progression-free survival of 12 months, while the overall survival in this scenario was 23 months. Conventional TACE had a progression-free survival of six months and an overall survival of 21 months, while for DEB-TACE, the parameters were seven months and 11 months, respectively. Patients who benefitted from RFA had a progression-free survival of 14 months and overall survival of 23 months. There was no disease-free survival for the advanced stages where patients received either sorafenib or the best supportive care. The overall survival was nine months for sorafenib and seven months for supportive care.

In patients who received a combination of therapies, the association of conventional TACE and sorafenib had an overall survival of 23 months and a progression-free survival of six months. When three treatment procedures were performed, the overall survival was 24 months, and disease-free survival was 11 months.

## Discussion

The presence of hepatocellular carcinoma was documented either using computed tomography (CT), magnetic resonance imaging (MRI), or contrast-enhanced ultrasonography (CEUS), in correlation with serum levels of alpha-fetoprotein. Liver biopsy was not performed for any of the patients due to its invasive character, susceptibility to associated complications, and the fact that imagistic methods and tumor markers were decisive in formulating the HCC diagnosis.

It is well known that the curative therapeutic options for patients with hepatocellular carcinoma are liver transplantation and liver resection [14,15]. While LT is the best choice for these patients, by treating both the tumor and the cirrhotic liver, its availability remains limited [14]. Within our study group, LT was possible in only two cases due to the low number of donors, with both patients having a disease-free survival of 12 months.

By comparison, liver resection is a more accessible solution but is recommended in patients with preserved hepatic function [15]. It has the advantage of eliminating the need to find a compatible allograft, but the risk of HCC recurrence is higher than in transplant recipients [14,16]. Current literature data showed that 70% of the patients who underwent surgical resection presented with HCC recurrence five years after resection [17]. One potential explanation is the fact that LR treats the tumor but does not have the capacity of stopping the progression of liver disease. Moreover, it is noticeable that, after interferon-free regimens were implemented, there have been many concerns regarding the relation between direct-acting antivirals and HCC recurrence. While several systematic reviews and meta-analyses concluded that DAAs do not affect in any way HCC recurrence [18-22], other studies have revealed a potential controversial association between interferon-free therapy and HCC, with DAAs resulting to have either promotive or suppressive effects, according to different authors [18,23-26]. Although data is conflicting, there is a certainty in the fact that HCC screening should not be stopped after achieving SVR, as antiviral treatment can cure the HCV infection, but not the liver disease itself. Within our study group, when analyzing patients with HCC previously treated with DAAs, surgical resection presented a one-year survival rate of approximately 71%, a value that seems to be in agreement with previous literature data concerning survival in patients with HCC.

The most frequently used bridging therapy before liver transplantation is TACE, either conventional or DEB-TACE [27]. In our study, the overall survival at one year in patients who benefitted from TACE was close to 80%.

Another therapeutic method used on the patients included in this study was RFA, a minimally invasive procedure that showed promising results in HCC management [15]. RFA works by generating heat, with the help of a high-frequency alternating current, leading to coagulative necrosis in tumoral cells and adjacent liver parenchyma [28]. Due to its availability, effectiveness, and minimally invasive character, RFA represents a first-line therapeutic option for early HCC [15]. Larger tumoral masses are, on the other hand, associated with a higher risk of recurrence after RFA than after surgical resection [28]. A possible explanation could be that large hepatomas may determine microvascular invasion, as well as micrometastases. Moreover, the heating resulting from RFA can lead to increased intra-tumor pressure, followed by the displacement of neoplastic cells through iatrogenic fistulae or shunts [28,29]. Compared to TACE, RFA usually requires fewer interventions and was found to be more efficient in terms of recurrence and survival [30,31]. However, in our study, the survival rate one year after ablation was of 69%, slightly lower than in patients who underwent TACE. It is important, though, to mention that the total number of patients undergoing RFA was almost four times lower than the patients undergoing TACE. We would also like to emphasize the possibility of using both therapeutic options, since clinical trials demonstrated that the combination of TACE and RFA has better survival outcomes than RFA alone in patients with nodules smaller than 7 cm [28,32]. Although our study included several patients with more than one therapeutic procedure, only one of them benefitted from both RFA and TACE, but that patient was submitted to a total of four therapeutic measures.

As expected, unresectable HCC is associated with poor prognosis, with a median survival of less than one year [33]. The survival rate at one year for patients in our study who received systemic chemotherapy was 24%. These patients also required hospitalization more frequently.

We observed that diabetes mellitus was more prevalent among patients who presented with disease progression, with previous research also suggesting that DM and insulin resistance are independent risk factors for HCC development [34,35]. Diabetes was also indicative of poor survival in our group. On the other hand, an improvement in survival has been noticed in patients who benefitted from multiple therapeutic procedures. When combining multiple therapeutic methods, the limitations of a certain procedure can be overcome by another intervention, thus being able to prevent HCC recurrence, decelerate progression, and reduce tumor size in these patients. For example, the use of postoperative TACE can block the nutrient vessels of the tumor, allowing the chemotherapeutic drug to kill the residual microscopic HCC cells, without producing damage to the normal liver cells. Combining TACE and RFA may increase coagulation necrosis, thus enabling effective treatment of larger masses than RFA alone may treat. We emphasize the fact that constant evaluation is of high importance in these patients while continuously searching for the best management option, as patients’ clinical status and biological parameters can quickly change. The purpose of all therapeutic measures applied to these patients (either strictly HCC-oriented or liver function supportive measurements) is either keeping patients inside the Milan criteria for liver transplantation or down-staging the disease so that transplant may be taken into consideration. In the management of patients diagnosed with HCV infection, a very important step is promptly removing the liver offender, as hepatic fibrosis is, at a certain point, capable of regression after eliminating the viral cause. At the same time, particular attention should be paid to HCC screening even after obtaining an SVR. Concerning our group of patients, all previously treated with direct-acting antivirals, we found that the various therapeutic methods applied for HCC were associated with outcomes similar to those already mentioned in the literature, regardless of antiviral treatment.

When liver functional reserve is adequate, the preferred therapy for localized HCC is represented by surgical resection. On the other hand, for individuals who are not eligible for resection (due to the extent of the tumor or due to the poor underlying liver status), the best option remains liver transplantation [36]. However, when considering liver transplantation as a therapeutic option, the availability of organs may be a major problem. When choosing the best therapeutic approach for a patient diagnosed with HCC, several factors must be taken into consideration, such as clinical status, liver function, oncological suitability, and organ availability. Each case has its own particularities, and the best therapeutic option must be chosen accordingly, as a universal approach for all patients does not exist.

It should be acknowledged that this study has several limitations, the primary limit being the low number of subjects observed. This aspect is due to the fact that we only included patients with hepatocellular carcinoma who were previously treated with direct-acting antivirals for hepatitis C virus infection. It is important to mention that, within our center, the proportion of patients who developed HCC after DAAs was low (at about 6%). Given the small study group and the fact that this was a single-center study, the results may not be generalizable. However, our findings regarding survival rates after HCC management in patients previously treated with DAAs are similar to those already reported in subjects with no history of direct-acting antiviral use. We encourage further research within this area, especially since literature data regarding HCC occurrence and recurrence after DAA treatment are conflicting. Larger prospective studies observing multicenter cohorts are required.

## Conclusions

So far, the best therapeutic option for hepatocellular carcinoma is liver transplantation. However, the low number of donors and the constant dropout risk (as a consequence of liver disease progression) may stand in the way of promptly performing this intervention. Patients may benefit from local procedures such as ablation or resection and especially a combination of these, as considered best suited for each patient. The ultimate goal of HCC treatment is to prolong survival, regardless of the chosen therapy. In our study, LR, TACE, and RFA significantly improved the survival of patients with unresectable HCC, but the benefit was higher when more than one therapeutic option was applied. Survival rates after HCC management in patients previously treated with DAAs are similar to those already reported in subjects with no history of direct-acting antiviral use.

## References

[1] Arnold M, Abnet CC, Neale RE, Vignat J, Giovannucci EL, McGlynn KA, Bray F (2020). Global burden of 5 major types of gastrointestinal cancer. Gastroenterology.

[2] Weaver AJ, Stafford R, Hale J, Denning D, Sanabria JR (2020). Geographical and temporal variation in the incidence and mortality of hepato-pancreato-biliary primary malignancies:1990-2017. J Surg Res.

[3] Villanueva A (2019). Hepatocellular carcinoma. N Engl J Med.

[4] Khatun M, Ray R, Ray RB (2021). Hepatitis C virus associated hepatocellular carcinoma. Adv Cancer Res.

[5] Andrei S, Isac S, Carstea M (2022). Isolated liver trauma: a clinical perspective in a non-emergency center for liver surgery. Exp Ther Med.

[6] Isac T, Isac S, Ioanitescu S (2021). Dynamics of serum α-fetoprotein in viral hepatitis C without hepatocellular carcinoma. Exp Ther Med.

[7] Serti E, Chepa-Lotrea X, Kim YJ (2015). Successful interferon-free therapy of chronic hepatitis C virus infection normalizes natural killer cell function. Gastroenterology.

[8] Abdelaziz AO, Nabil MM, Abdelmaksoud AH (2019). Tumor behavior of hepatocellular carcinoma after hepatitis C treatment by direct-acting antivirals: comparative analysis with non-direct-acting antivirals-treated patients. Eur J Gastroenterol Hepatol.

[9] El Kassas M, Elbaz T, Salaheldin M, Abdelsalam L, Kaseb A, Esmat G (2019). Impact of treating chronic hepatitis C infection with direct-acting antivirals on the risk of hepatocellular carcinoma: the debate continues - a mini-review. J Adv Res.

[10] Reig M, Forner A, Rimola J (2022). BCLC strategy for prognosis prediction and treatment recommendation: the 2022 update. J Hepatol.

[11] Johnson PJ, Berhane S, Kagebayashi C (2015). Assessment of liver function in patients with hepatocellular carcinoma: a new evidence-based approach-the ALBI grade. J Clin Oncol.

[12] Oken MM, Creech RH, Tormey DC, Horton J, Davis TE, McFadden ET, Carbone PP (1982). Toxicity and response criteria of the Eastern Cooperative Oncology Group. Am J Clin Oncol.

[13] Lencioni R, Llovet JM (2010). Modified RECIST (mRECIST) assessment for hepatocellular carcinoma. Semin Liver Dis.

[14] Koh JH, Tan DJ, Ong Y (2022). Liver resection versus liver transplantation for hepatocellular carcinoma within Milan criteria: a meta-analysis of 18,421 patients. Hepatobiliary Surg Nutr.

[15] Portolani N, Coniglio A, Ghidoni S, Giovanelli M, Benetti A, Tiberio GA, Giulini SM (2006). Early and late recurrence after liver resection for hepatocellular carcinoma: prognostic and therapeutic implications. Ann Surg.

[16] Menahem B, Lubrano J, Duvoux C (2017). Liver transplantation versus liver resection for hepatocellular carcinoma in intention to treat: an attempt to perform an ideal meta-analysis. Liver Transpl.

[17] Marín-Hargreaves G, Azoulay D, Bismuth H (2003). Hepatocellular carcinoma: surgical indications and results. Crit Rev Oncol Hematol.

[18] Miuma S, Miyamoto J, Taura N (2020). Influence of interferon-free direct-acting antiviral therapy on primary hepatocellular carcinoma recurrence: a landmark time analysis and time-dependent extended Cox proportional hazards model analysis. Intern Med.

[19] Waziry R, Hajarizadeh B, Grebely J (2017). Hepatocellular carcinoma risk following direct-acting antiviral HCV therapy: a systematic review, meta-analyses, and meta-regression. J Hepatol.

[20] Manthravadi S, Paleti S, Pandya P (2017). Impact of sustained viral response postcurative therapy of hepatitis C-related hepatocellular carcinoma: a systematic review and meta-analysis. Int J Cancer.

[21] Singh S, Nautiyal A, Loke YK (2018). Oral direct-acting antivirals and the incidence or recurrence of hepatocellular carcinoma: a systematic review and meta-analysis. Frontline Gastroenterol.

[22] Li DK, Chung RT (2019). Overview of direct-acting antiviral drugs and drug resistance of hepatitis C virus. Methods Mol Biol.

[23] Konjeti VR, John BV (2018). Interaction between hepatocellular carcinoma and hepatitis C eradication with direct-acting antiviral therapy. Curr Treat Options Gastroenterol.

[24] Warzyszyńska K, Jonas M, Wasiak D, Kosieradzki M, Małkowski P (2017). Accelerated hepatocellular carcinoma recurrence rate after postoperative direct-acting antivirals treatment - preliminary report. Clin Exp Hepatol.

[25] Villani R, Facciorusso A, Bellanti F (2016). DAAs rapidly reduce inflammation but increase serum VEGF level: a rationale for tumor risk during anti-HCV treatment. PLoS One.

[26] Mashiba T, Joko K, Kurosaki M (2018). Does interferon-free direct-acting antiviral therapy for hepatitis C after curative treatment for hepatocellular carcinoma lead to unexpected recurrences of HCC? A multicenter study by the Japanese Red Cross Hospital Liver Study Group. PLoS One.

[27] Majno PE, Adam R, Bismuth H (1997). Influence of preoperative transarterial lipiodol chemoembolization on resection and transplantation for hepatocellular carcinoma in patients with cirrhosis. Ann Surg.

[28] Zhang YJ, Chen MS, Chen Y, Lau WY, Peng Z (2021). Long-term outcomes of transcatheter arterial chemoembolization combined with radiofrequency ablation as an initial treatment for early-stage hepatocellular carcinoma. JAMA Netw Open.

[29] Kang TW, Lim HK, Lee MW (2015). Aggressive intrasegmental recurrence of hepatocellular carcinoma after radiofrequency ablation: risk factors and clinical significance. Radiology.

[30] Cho YK, Kim JK, Kim MY, Rhim H, Han JK (2009). Systematic review of randomized trials for hepatocellular carcinoma treated with percutaneous ablation therapies. Hepatology.

[31] Weis S, Franke A, Mössner J, Jakobsen JC, Schoppmeyer K (2013). Radiofrequency (thermal) ablation versus no intervention or other interventions for hepatocellular carcinoma. Cochrane Database Syst Rev.

[32] Peng ZW, Zhang YJ, Chen MS (2013). Radiofrequency ablation with or without transcatheter arterial chemoembolization in the treatment of hepatocellular carcinoma: a prospective randomized trial. J Clin Oncol.

[33] Cabibbo G, Enea M, Attanasio M, Bruix J, Craxì A, Cammà C (2010). A meta-analysis of survival rates of untreated patients in randomized clinical trials of hepatocellular carcinoma. Hepatology.

[34] Fujita K, Iwama H, Miyoshi H (2016). Diabetes mellitus and metformin in hepatocellular carcinoma. World J Gastroenterol.

[35] Stefan N, Häring HU, Cusi K (2019). Non-alcoholic fatty liver disease: causes, diagnosis, cardiometabolic consequences, and treatment strategies. Lancet Diabetes Endocrinol.

[36] Kow AW (2019). Transplantation versus liver resection in patients with hepatocellular carcinoma. Transl Gastroenterol Hepatol.

